# Glucose Metabolic Characterization of Human Aqueous Humor in Relation to Wet Age-Related Macular Degeneration

**DOI:** 10.1167/iovs.61.3.49

**Published:** 2020-03-30

**Authors:** Guoge Han, Pinghui Wei, Meiqin He, He Teng

**Affiliations:** 1 Tianjin Eye Hospital, Tianjin Key Lab of Ophthalmology and Visual Science, Tianjin, P.R. China; 2 Eye Institute and School of Optometry and Ophthalmology, Tianjin Medical University Eye Hospital, Tianjin, P.R. China

**Keywords:** aqueous humor, glucose metabolism, age-related macular degeneration

## Abstract

**Purpose:**

Energy compromise underpins wet age-related macular degeneration (wAMD) pathogenesis, but the relationship between glucose metabolism and the disease remains unclear. Here, we characterized aqueous humor (AH) to elucidate glucose-related metabolic signatures in patients with wAMD.

**Methods:**

In total, 25 eyes of 25 patients with wAMD were divided into phakic (15 eyes), pseudophakic (10 eyes), and intravitreal injections of ranibizumab (13 eyes) wAMD groups. Twenty patients with cataract (21 eyes) served as controls. Ultrahigh-performance liquid chromatography tandem mass spectrometry was used to quantitatively characterize AH.

**Results:**

Twenty-one metabolites related to glucose metabolism were identified in AH from 45 patients. Tricarboxylic acid (TCA)-related metabolic substrates, including citrate, were detected in AH and were significantly increased in AMD (*P* < 0.01) and AMD pseudophakic groups (*P* < 0.05). In contrast, *α*-ketoglutarate levels were decreased in the AMD group (*P* < 0.05). The *α*-ketoglutarate/citrate ratio was significantly decreased, corresponding to 71.71% and 93.6% decreases in the AMD (phakic and pseudophakic) groups as compared with controls (*P* < 0.001), revealing a significant positive correlation with glutamine. A lower mean glutamine and higher glutamate level were detected in AMD cases compared with controls. No significant differences were observed for lactic acid or other Krebs cycle metabolites. Intravitreal injection significantly alleviated mean central foveal thickness but did not significantly alter metabolites.

**Conclusions:**

Compromised glucose TCA cycle and altered glutamine metabolism are implicated in the AH metabolism in wAMD. These findings highlight potential treatments for alleviating wAMD from a metabolic perspective.

Age-related macular degeneration (AMD) is becoming the leading cause of aging blindness and the third cause of adult blindness in developing countries.^1^ The World Health Organization predicted that global AMD prevalence will reach 288 million by 2040.^2^ AMD patients may progress to advanced disease, including choroidal neovascularization (CNV).^3^ Ultimately, this disease may lead to central vision loss, which then affects quality of life in the elderly.^4^ AMD pathogenesis remains unclear and is regarded as multifactorial, with mitochondrial genetic deficiency and metabolic compromise interacting with environmental factors.^5^

The human retina is one of the highest energy-consuming and metabolically active tissues; it is assumed that these metabolic and energy needs are mainly met by glucose.^6^ Although the retina is part of the brain, its energy metabolism pattern has greater similarities with that of cancerous tissue: both tissues preferentially produce the majority of their energy via aerobic glycolysis (the Warburg effect), whereby pyruvate is preferably converted to lactate rather than metabolized via the tricarboxylic acid (TCA) cycle.^7^^–^^9^ Previous work in vitro has indicated that glucose is metabolized to a greater degree via the glycolytic pathway in a mitochondrial injury model.^10^ Similarly, Joyal et al.^11^ pointed out that retinal energy metabolism dysregulation might be a driving force in neovascular AMD development. In exudative AMD, CNV formation is associated with activated glycolytic enzymes and influencing glucose metabolism.^12^ It is therefore essential to be able to detect substances related to glucose energy metabolism in human AMD biofluid samples.

Energy metabolites are the products of the upstream cumulative effects of glycolysis or TCA-related genes, proteins, and their interactions with pathogenic environmental factors.^13^ Thus quantification of metabolites is thought to reflect the true functional states of disease phenotypes, such as AMD.^14^ Biofluid markers (plasma, serum, and urine) of AMD incidence and progression have been investigated, mainly in relation to inflammation, complement, and lipid metabolism.^15^^–^^18^ Although promising, this research has only provided serologic biomarkers, which may be affected by dietary patterns of patients and general dynamics of metabolic parameters.^15^ Thus it is challenging to elucidate intraocular metabolic states through serologic tests.

Aqueous humor (AH) is secreted into the posterior chamber of the eye by nonpigmented epithelial ciliary processes. Facing the vitreous, AH acts as a reservoir harboring growth factors and regulating mediators contributing to AMD pathogenesis.^19^ Alterations in AH proteins and cytokines are observed in anterior segment disorders and retinal diseases.^20^^,^^21^ Thus AH analyses may be valuable for understanding the mechanisms underlying retinal disorders. In addition, the use of anti-VEGF (vascular endothelial growth factor) therapy from a metabolic perspective remains unexplored.

The current study aimed to characterize the AH glucose metabolic signatures of patients with AMD and to compare metabolic changes after anti-VEGF intravitreal injections using ultrahigh-performance liquid chromatography tandem mass spectrometry (UHPLC-MS/MS). Ultimately, we sought to investigate the role of glucose metabolism in patients with AMD and the underlying mechanisms by which the eye counteracts side effects in the presence of ischemic injury. A deeper understanding of atypical retinal energy metabolism and its response to anti-VEGF treatment will shed light on disease pathogenesis and provide relevant translational information.

## Methods

### Study Design

This was a prospective case–control study performed in the Tianjin Eye Hospital. The study was approved by the Tianjin Eye Hospital Ethics Committee and adhered to the tenets of the Declaration of Helsinki. It was also registered online with the Chinese Clinical Trial Registry (ChiCTR1900022442). All included participants provided written informed consent prior to commencement of the study.

### Human Subjects

Patients with a diagnosis of wet age-related macular degeneration (wAMD) at the time of outpatient appointments from April to June 2019 were included in this study. Because the crystalline lens may also contribute to AH metabolic signatures, the AMD group was subdivided into AMD phakic and pseudophakic groups. AH specimens were collected during the initial intravitreal ranibizumab (IVR; 0.5 mg) injection. AMD intravitreal injection (post-IVR sample) was performed on the second round of IVR (1 month after the initial IVR treatment). The control group was composed of age-matched patients undergoing cataract surgery without AMD.

All wAMD participants underwent a comprehensive eye examination, including the measurement of uncorrected distant visual acuity, corrected distant visual acuity, subjective refraction, noncontact tonometry, slit-lamp examination, dilated fundus examination, spectral domain optical coherence tomography (OCT), fluorescein angiography, and indocyanine green angiography.

Exclusion criteria included the presence of any retinopathy other than AMD, uveitis or ocular infection, glaucoma, corneal diseases, significant opacities, refractive error of 6 diopters or more of spherical equivalent, past history of any other ocular surgery or intraocular procedure (such as laser or intravitreal injections), and/or diabetes mellitus.

### Sample Collection

All AH samples were collected before intravitreal injection or cataract surgery in the operating room using an operating microscope. Undiluted samples were transferred into sterile containers. To remove debris, samples were clarified by centrifugation in sterile tubes at 15,000 *g* for 5 minutes and stored immediately in a −80°C freezer without thawing prior to analysis.

### UHPLC-MS Analysis

Frozen AH samples were thawed and subjected to UHPLC-MS. The liquid chromatography tandem mass spectrometry (LC/MS) portion of the platform was based on a Thermo Fisher Vanquish UHPLC (Waltham, MA, USA) equipped with an ACQUITY UHPLC BEH Amide column (1.7 µm, 2.1 mm × 100 mm; Waters Corp., Milford, MA, USA) and Thermo-TSQ Vantage (Thermo Fisher) mass spectrometer. Metabolites were detected in electrospray negative- and positive-ionization mode. Samples were injected sequentially into a Thermo-TSQ Vantage mass spectrometer equipped with a Vanquish UHPLC system with autosampler (Thermo Fisher). The ACQUITY UPLC BEH Amide column was then heated to 45°C under a flow rate of 300 µL/min. A gradient was used to separate the compounds consisting of 20 mM ammonium acetate (solvent A) and 5% acetonitrile (solvent B). The gradient started at 5% solvent A for 1 minute, increased linearly to 35% solvent A over 13 minutes, and then increased linearly to 60% solvent A over 2 minutes with a 2-minute hold before returning to the starting mixture for 0.1 minute and re-equilibrating for 4 minutes. Quality control (QC) samples were injected every six or eight samples during acquisition.

MS conditions were as follows: collision gas pressure (mTorr): 1.0; Q1 peak width (FWHM): 0.70; Q3 peak width (FWHM): 0.70; cycle time (s): 1.500; capillary temperature: 350.0°C; vaporizer temperature: 350.0°C; sheath gas pressure: 35.0; aux valve flow: 10.0; spray voltage: positive polarity –3500.0 V; negative polarity –3000.0 V; scan type: selected reaction monitoring/multiple reaction monitoring. A standard dilution containing 21 metabolites was prepared for LC-MS analysis. The peak area of each standard metabolite was prepared from serially diluted reference standard solutions with the corresponding concentration. Calibration curves were constructed using the least-squares method.

To ensure data quality for metabolic profiling, QC samples were prepared by pooling aliquots representative of all samples under analysis and were later used for data normalization. QC samples were prepared and analyzed with the same procedure that was used for experimental samples in each batch. Metabolites with QC-RSD (relative standard deviation) less than 30% were regarded as accepted data QC in terms of stability and reliability. Dried extracts were dissolved in 50% acetonitrile. Each sample was filtered with a disposable 0.22 µm cellulose acetate membrane, transferred into 2 mL UHPLC vials, and stored at –80°C until analysis.

### Data Preprocessing and Filtering

Raw selected reaction monitoring data files were processed by peak finding, alignment, and filtering using Xcalibur Qual browser software (Thermo Fisher). The concentration of each metabolite was calculated from the calibration curve of corresponding standard metabolites.

### Statistical Analysis

Statistical analysis was performed using the SPSS software (GLM Univariate, version 21, IBM Corp., Armonk, NY, USA). The independent *t*-test was used to compare the means of continuous variables between groups. All data were tested for Gaussian distribution. Results are expressed as means ± SEM. The Pearson correlation coefficients were computed to investigate linear relationships between variables. *P* values <0.05 were considered statistically significant.

## Results

### Study Population

We recruited 45 participants, 44.4% (*n* = 20) with normal macular health (control group) and 55.6% (*n* = 25) with AMD. Table 1 presents the clinical and demographic characteristics of the study group. Among the potential confounders evaluated, no significant difference could be observed among the different study groups.

**Table 1.** General Information and Clinical Characteristics

Characteristic	Controls (*n* = 20)	AMD (*n* = 25)	*P* Value
Age, y	70.1	72.09	0.408
Fasting blood sugar, mmol/L	5.82	5.61	0.350
Female, %	35	52.17	0.258
Alcohol, %	5	8.70	0.635
Smokers, %	10	21.74	0.531
Hypertension, %	45	52.17	0.639
Hyperlipidemia, %	10	8.70	0.883
Cerebral infarction, %	5	21.74	0.255
Coronary heart disease, %	10	26.09	0.337

General information and clinical characteristics were compared between AMD and control cataract patients using a *t*-test for continuous variables, and the χ2 test for categorical variables. The mean age and mean blood sugar values are presented in the chart.

### UHPLC-MS Analysis

**Figure 1.**
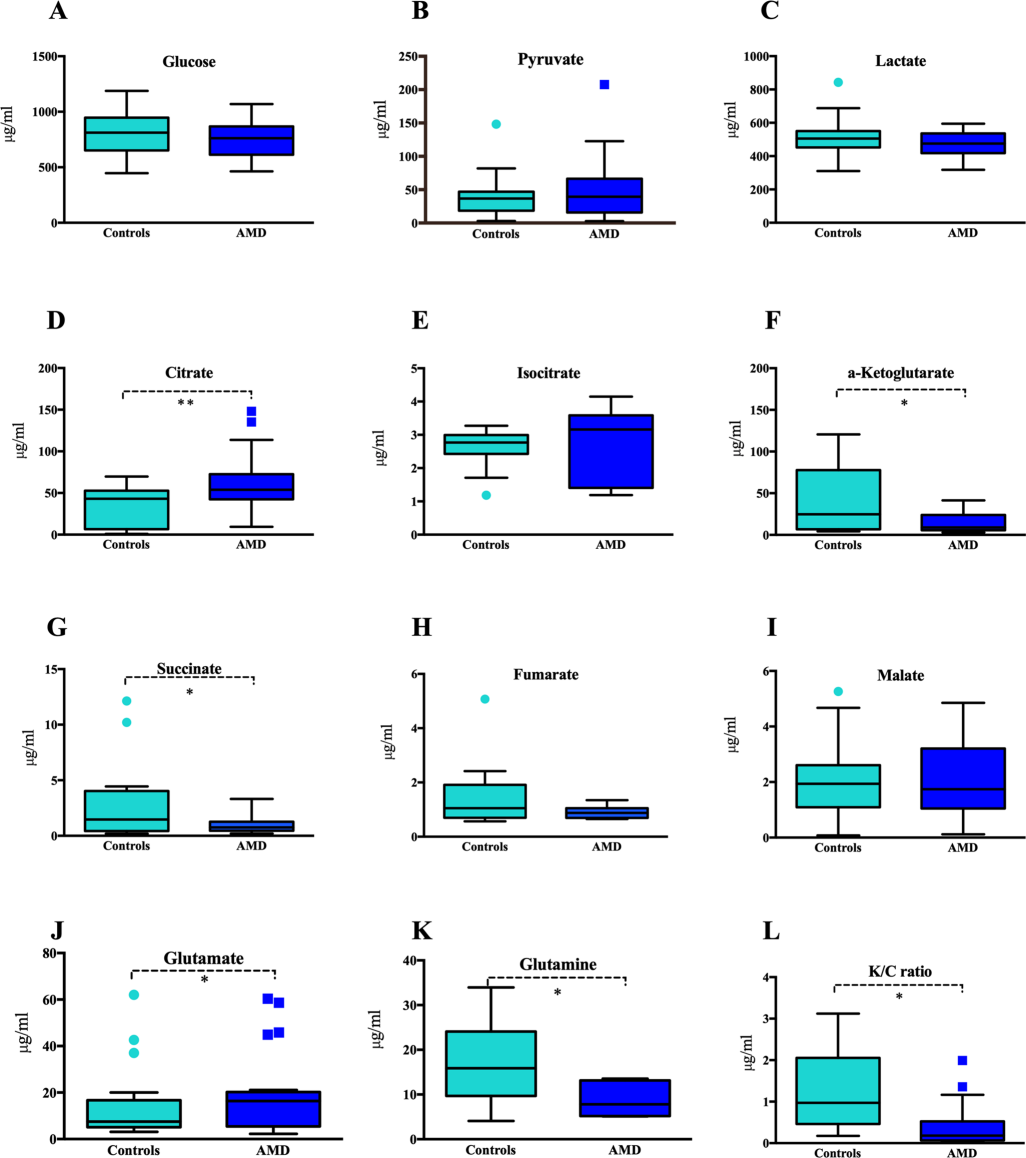
Concentration of glucose metabolism–related subtracts in AH verified by UHPLC-MS/MS between control and AMD groups. Metabolites related to glucose metabolism were identified (Figs. A-L), and most were absolute quantified in AH analyzed samples, and box plots are summarized in the figure. The comparison between control (*n* = 21) and wAMD groups (*n* = 25) highlighted significantly different molecules (*P* < 0.05). *Statistically significant (*P* < 0.05).

Twenty-one metabolites related to glucose metabolism were identified in AH samples. Results (including *P* values and box plots) are summarized in Figure 1. Detailed AH specimen preparation, LC conditions, and MS parameters are shown in Supplementary Table S1. Prior to statistical analysis, QC was performed to exclude metabolites with QC-RSD in more than 30% of samples (Supplementary Fig. S1).

In both control and AMD groups, endogenous metabolites from AH could be separated well and annotated by comparison with corresponding standards. Peak area ratios of targeted compounds to internal standards were recalculated for *t*-test analysis. As for TCA-related metabolites, citrate, isocitrate, succinate, and *α*-ketoglutarate were detected in AH and were significantly different in the AMD group (*P* < 0.05; Fig. 1). Citrate (70.26 ± 7.12 µg/mL vs. 33.89 ± 6.22 µg/mL) and isocitrate (3.55 ± 0.18 µg/mL vs. 2.63 ± 0.19 µg/mL) were significantly increased in the AMD group, whereas *α*-ketoglutarate (12.50 ± 2.40 µg/mL vs. 42.18 ± 13.05 µg/mL) and succinate (0.92 ± 0.17 µg/mL vs. 4.82 ± 1.55 µg/mL) were significantly decreased compared with control levels. Previous studies have revealed that glutamine is a major cellular carbon source during hypoxia or mitochondrial impairment.^22^ Glutamine (6.61 ± 0.76 µg/mL vs. 18.35 ± 3.70 µg/mL) was decreased in the AH of patients with AMD, whereas glutamate (11.99 ± 1.52 µg/mL vs. 7.60 ± 1.01 µg/mL) was increased (*P* < 0.05; Figs. 1J, 1K).

### Correlation Between Targeted Metabolites and General Ocular Factors or General Characteristics

**Figure 2.**
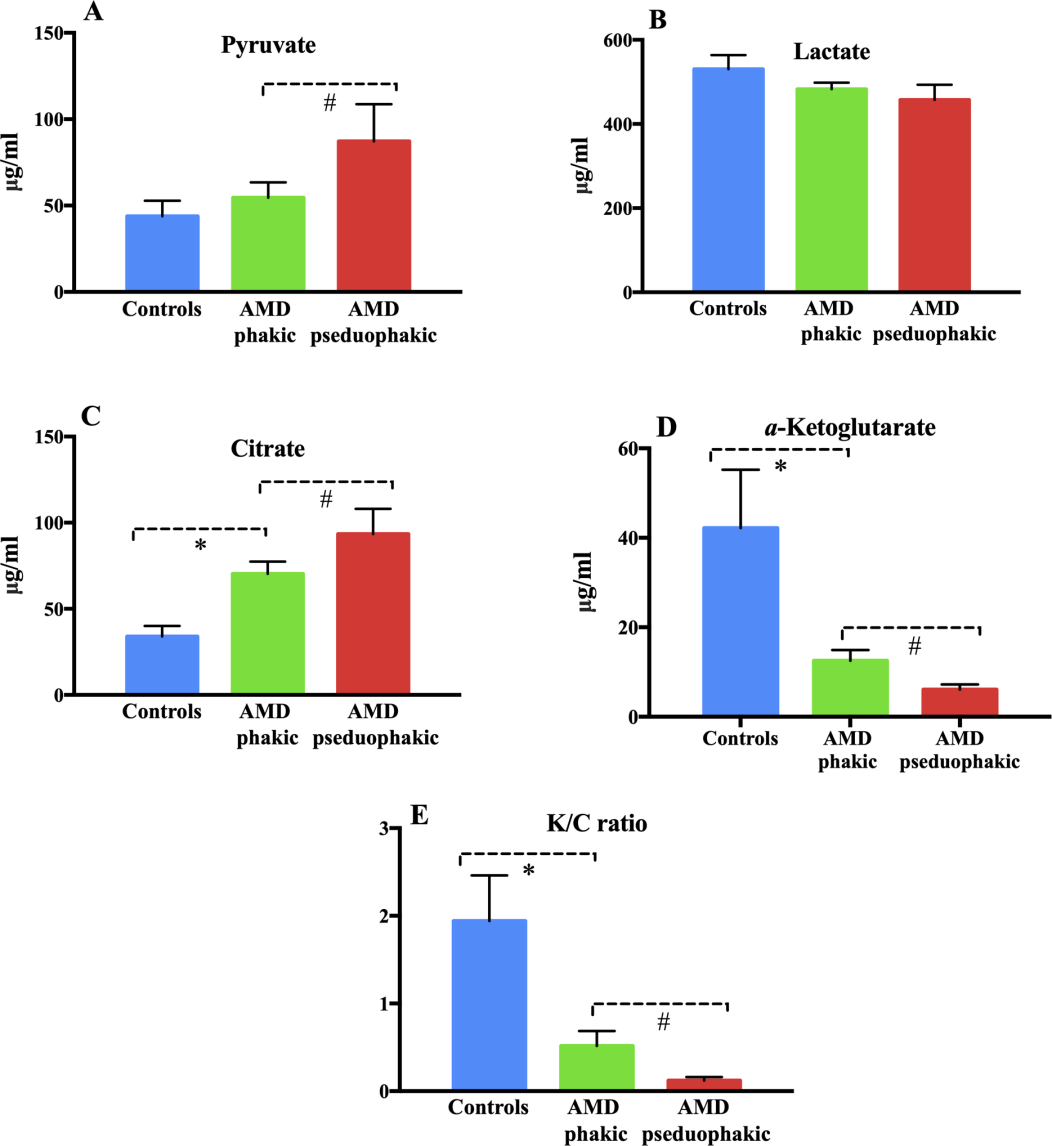
Analysis of the correlation between detected metabolites and crystalline lens form concentrations of citrate, pyruvate, lactate, and *α*-ketoglutarate and K/C ratio in AH from the AMD phakic and AMD pseudophakic groups. The mean pyruvate level of AH was significantly decreased in the AMD phakic group when compared with the AMD pseudophakic group (*P* < 0.05) (**A**). Additionally, the AH lactic acid concentration of the AMD phakic group shows no significant difference with the AMD pseudophakic group and the control group (*P* > 0.05) (**B**). The mean citrate level of the AH was significantly elevated in the AMD phakic group when compared with the AMD pseudophakic group (*P* < 0.01) (**C**). The mean α-ketoglutarate level of the AH was significantly decreased in the AMD phakic group when compared with the AMD pseudophakic group (*P* < 0.01) (**D**). The K/C ratio of the AMD pseudophakic group was also evidently lower than that of the phakic group (*P* < 0.001) (**E**). *#Statistically significant (*P* < 0.05).

To investigate the contribution of lens glucose metabolism to AH in the AMD group, we analyzed correlations between altered metabolites determined by UHPLC-MS/MS and form of crystalline lens (phakic or pseduophakic eye). Pyruvate and citrate levels in the AH of AMD phakic patients were significantly lower than those in the pseudophakic AMD group (*P* < 0.05; Figs. 2A, 2C). The *α*-ketoglutarate concentration was significantly higher in AMD phakic patients than in the intraocular lens AMD group (*P* < 0.05; Fig. 2D). By contrast, lactic acid showed no significant difference between the control and phakic or pseudophakic AMD groups (*P* > 0.05; Fig. 2B). No significant correlations were observed for other independent factors (age, sex, and systemic factors).

### *α*-Ketoglutarate/Citrate (K/C) Ratio in AH

Cellular conditions that increase the K/C ratio initiate reductive glutamine metabolism, particularly in cancer cells.^23^ We quantitatively assessed the K/C ratio in AH with UHPLC-MS analysis. Compared with cataract controls, AMD eyes displayed significantly lower (*P* < 0.001) ratio of K/C (Fig. 1L). Further subgroup AH analysis revealed that the K/C ratio of the AMD pseudophakic group was also evidently lower than that of the phakic group (*P* < 0.001; Fig. 2E).

**Figure 3.**
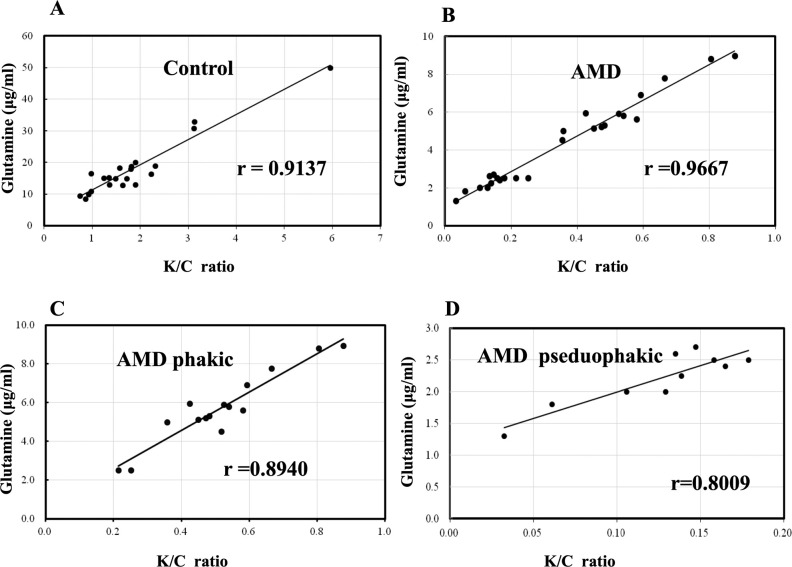
Correlation analysis of K/C ratio and glutamine in the AH from control and AMD patients. The Pearson correlation coefficients were computed to investigate the relationship between K/C and glutamine from the control group (**A**) and the AMD group (**B**). Correlation of K/C ratio and glutamine in the AH from AMD phakic and AMD pseduophakic patients are shown in (**C**, **D)**.

To gain insight into the contribution of other factors (age, sex, and general status) on K/C ratio in AMD, we evaluated the correlation between these factors with the K/C ratio. No significant differences were observed in both groups (*P* > 0.05).

### Correlation with Glutamine in the AH of Control and AMD Eyes

Analysis of the K/C ratio revealed a significant positive correlation with glutamine in the AH of control eyes (r = 0.9137, *P* < 0.01; Fig. 3A) and in the AMD group (r = 0.9667, *P* < 0.01; Fig. 3B). Also, the K/C ratio in both the AMD phakic and pseduophakic groups revealed a significant correlation with glutamine (r = 0.8904, *P* < 0.001; Fig. 3C) (r = 0.8009, *P* < 0.001; Fig. 3D).

### Effects of IVR Injection on AH Metabolite Levels in Patients with AMD

No identified metabolites in AH samples were significantly changed after IVR (*P* > 0.05, Table 2). OCT showed a tendency of decrease in central macular thickness after IVR treatment (*P* < 0.05, Table 2).

**Table 2.** Changes in AH Metabolites Related to Glucose Metabolism Before and After IVR

Metabolites	Pre-IVR (µg/mL)	Post-IVR (µg/mL)	*t*	*P*
Glucose	652.80 ± 138.96	631.42 ± 129.03	1.684	0.191
Pyruvate	36.96 ± 5.93	39.53 ± 5.69	–0.477	0.643
Lactate	482.23 ± 21.23	498.69 ± 26.81	–0.736	0.478
Citrate	79.63 ± 14.05	64.20 ± 8.78	0.898	0.404
Isocitrate	2.63 ± 0.19	3.55 ± 0.18	–0.007	0.402
α-Ketoglutarate	11.31 ± 2.78	19.17 ± 6.43	–1.199	0.276
Succinate	0.97 ± 0.31	0.99 ± 0.39	–0.083	0.935
Fumarate	2.04 ± 0.55	0.85 ± 0.09	0.104	0.233
Malate	1.93 ± 0.51	2.02 ± 0.50	–0.174	0.865
K/C ratio	0.30 ± 0.05	0.32 ± 0.10	–1.261	0.454
Glutamine	18.35 ± 3.70	14.61 ± 0.76	1.026	0.521
Glutamate	15.63 ± 2.14	9.80 ± 2.40	1.827	0.117
AMP	0.011 ± 0.002	0.016 ± 0.007	–0.010	0.100
cAMP	0.0007 ± 0.0001	0.0011 ± 0.0002	–0.611	0.618
TMP	0.26 ± 0.04	0.18 ± 0.06	0.440	0.536
1PG	2.80 ± 0.38	2.18 ± 0.18	0.005	0.219
IMP	0.18 ± 0.01	0.14 ± 0.02	0.022	0.545
FMN	4.50 ± 1.15	3.34 ± 1.60	0.559	0.596
Adenosine	1.65 ± 0.34	2.18 ± 1.34	0.718	0.493
NAD	0.036 ± 0.007	0.045 ± 0.011	–0.059	0.090
CMT	401 ± 43.28	287.46 ± 16.63	2.545	0.026*

Changes in AH metabolites related to the Warburg effect before and after IVR. AMP, adenosine monophosphate; cAMP, cyclic adenosine monophosphate; TMP, trimethoprim; 1PG, D-Glucose 1-phosphate; IMP, inosine 5'-monophosphate; FMN, flavin mononucleotide; NAD, nicotinamide adenine dinucleotide; CMT, central macular thickness. *Statistically significant (*P* < 0.05).

## Discussion

Here we present a cross-sectional observation of glucose-related metabolites in AH specimens from wAMD and control cataract groups. Using UHPLC-MS/MS analysis, we further observed significant differences in the levels of TCA metabolites from both crystalline lens (phakic or pseduophakic eyes). We also observed a higher K/C ratio in the AH of patients with cataract and a lower K/C ratio in patients with AMD. The other potential confounding effects of sex, age, and general characteristics on these results were negligible. Despite the relatively small cohort of subjects with a single subtype of wAMD, to our knowledge, this study presents the first characterization of glucose-related metabolites in AH.

Energy compromise is part of the pathogenesis underlying AMD.^24^ In recent years, the Warburg effect has become an active area of study within the cancer and eye research community, provided through a deeper understanding of the Warburg effect at the molecular level.^25^^–^^27^ However, similarities between the human AMD eye and in vitro experiments are unexplored. Therefore a better understanding of human retinal energy metabolism in normal and AMD disease is of great importance. Osborn et al.^28^ first applied MS plasma metabolomics from AMD (CNV) patients primarily related to peptides and modified amino acids, exhibiting higher levels of pyruvate and lower levels of glutamine intermediates in AMD. Although certain TCA-related organic acids were analyzed in the samples from Osborn et al.,^28^ the putative metabolite identifications still need to be confirmed by LC/MS and authentic standard samples.^29^

Our AH quantitative analysis of AMD fingerprints, with particular interest in glucose-related metabolites, presented both similarities and differences with previous articles. First, AH showed no significant increase in lactate concentrations, which is the preferable production of aerobic glycolysis in the retina.^30^ As epithelial crystalline lens cells also produce lactate, we recruited a pseduophakic AMD group to avoid metabolic interference from lens metabolism. As a result, there was a decrease in lactate in intraocular lens AMD participants compared with that in phakic AMD patients, although no significant difference could be observed. Theoretically, lactate in the retinae does not fully diffuse into the AH; instead, it tends to accumulate in tissues where oxygen supply is insufficient.^31^^,^^32^ In phakic patients, the crystalline lens may act as a physical or metabolic barrier.^33^ This implies that in patients with AMD, lactate possibly accumulates in the retina or surface nearby the macular. Further experiments investigating lactic acid distribution between the AH, vitreous, and retina in CNV animal models are necessary to confirm this hypothesis. Considering that lactate is affected by multifactors, such as lens barrier, ciliary body secretion, and cornea, as well as general status, lactic acid may not be suitable for potential AMD biomarker candidates. However, investigating its metabolism mechanism is helpful in elucidating the AH dynamic change of glucose and aerobic glycolysis in human clinical trials.

Second, compared with control subjects, AH pyruvate concentrations were elevated significantly in pseudophakic (*P* < 0.05) but not phakic patients (Fig. 2). This is possibly due to decreased fuel consumption from the epithelial crystalline cells in the anterior chamber.^34^ To our knowledge, there is only one previous report that used a metabolomics approach in describing both glucose and pyruvate concentrations in living human subjects from plasma.^15^ Our own AH findings from patients with AMD were consistent with this report.

The observation of increased citrate concentration of AH in AMD phakic and pseudophakic groups supports previous results showing that citrate may act as a key mediator in metabolic reprogramming rather than merely a process for energy production.^35^ Citrate is produced in the Krebs cycle from acetyl-CoA and converted to *α*-ketoglutarate or activated acetyl-CoA carboxylase for the purpose of fatty acid biosynthesis.^36^ In our study, *α*-ketoglutarate concentration from AH was noticeably decreased regardless of form. Low *α*-ketoglutarate levels promote the stabilization of hypoxia-inducible factor 1*α* (HIF-1*α*) and secretion of VEGF-A, leading to macular neovascularization.^6^ Thus consistent with previous suggestions, dysregulation of glucose metabolism, particularly in TCA cycles, may be a driving force in AMD and other retinal disease pathogenesis, but the underlying mechanisms require further exploration.^6^^,^^37^^,^^38^

The decrease in glutamine levels in AH in AMD participants was unsurprising, given that published articles on AMD metabolomics demonstrate higher plasma levels of glutamine in early AMD^39^ and lower levels in intermediate AMD.^29^ However, Hu et al.^40^ proposed that the lens may be involved in contributing compounds to the AH, thereby maintaining glutamine homeostasis in ischemia and reperfusion models. As nonpigmented epithelium cells of ciliary epithelium reduce glutamine efflux into the AH in injury models, dysfunction of the uptake transporter in the lens or reversal of the system leading to glutamine efflux from the lens ultimately causes glutamine to remain in the AH.^40^^,^^41^ In the current study, we observed a slight elevation in glutamine concentration in the AMD pseudophakic group compared with that in phakic AMD controls. This result may indicate that glutamine was in shortage regardless of lens form in AH of patients with AMD.

Notably, we identified a high K/C ratio in the AH of patients with age-related cataract and a lower K/C ratio in AMD participants. This ratio revealed strong positive correlations between glutamine in all groups. In standard growth conditions, proliferation-relevant fatty acids are barely produced from glutamine.^23^ During hypoxia or mitochondrial inhibition, glutamine conversion from oxidative to reductive is regarded as a major carbon source of fatty acid synthesis.^42^^,^^43^ This particular switch was of great importance for sustaining rapid cell proliferation and was triggered by any event responsible for alterations in the K/C ratio.^23^ Our observation of K/C ratio (1.94 ± 0.42) in control patients possibly was in agreement with previous conceptions that lens tissue exhibits a high dependence on aerobic glycolysis, owing to the loss of organelles, including mitochondria in lens fibers. Mitochondrial inhibition in the anterior chamber possibly resulted in reduced metabolism of glutamine as an essential carbon source for proliferating cells. Other anterior chamber tissue, such as cornea and the ciliary epithelium, may also contribute to the higher K/C ratio in AH, but this hypothesis needed to be validated in further experiments. In contrast, in the AMD group, K/C ratio was evidently decreased (0.23 ± 0.06), accompanied by a significant decline in glutamine (r = 0.9667, *P* < 0.01). To minimize lens influence on K/C ratio alterations, the AMD pseudophakic group was compared with controls, displaying a lower K/C ratio (0.08 ± 0.03). Several reasons may explain the relatively lower K/C ratio in the pseduophakic group. First, in lens the citric acid cycle only occurs in the epithelium because these are the only lens tissues that possess mitochondria.^6^ Artificial lens patients are lacking a portion of epithelial cells, which leads to the accumulation of higher concentration of citrate in the AH. Adding that *α*-ketoglutarate concentration was downregulated regardless of lens form in the AMD group as we discussed earlier, this decrease may promote the stabilization of HIF-1*α* and secretion of VEGF-A, contributing to macular neovascularization, as well as relatively lower K/C ratio.^6^ Another possible mechanism may include the normal retinal pigment epithelium (RPE) cells that have a high rate of reductive carboxylation, whereby the glutamine enters the TCA with generation of citrate, displaying a higher K/C ratio for fatty acid biosynthesis.^6^^,^^44^ In AMD, the ageing and apoptosis of RPE cells, leading to a disrupted redox balance and impairment of fatty acid synthesis,^6^^,^^45^ are possibly correlated with the decreases in reductive glutamine use, showing lower levels of K/C. However, this hypothesis still needs to be validated from animal models with isotope labeled experiments or further clinical trials. In addition, although anti-VEGF treatment alleviated macular edema and enhanced visual acuity, it failed to alter the K/C ratio and other metabolites in this trial. This suggested that replenishing glutamine is promising as a future AMD therapeutic target (Table 2). New treatments may require an approach that impacts intrinsic properties of the metabolic network, rather than mere interruption of a signaling pathway.

Theoretically, the average age of patients subjected to cataract surgery in China is supposed to be younger than that of wAMD patients with intravitreal anti-VEGF injection, due to different onset time of the two diseases and medical health insurance reasons. Thus the study was strictly designed and conducted according to age-matched inclusion criteria because these factors are believed to affect general or localized glucose metabolism significantly. Other factors as shown in Table 1, the AMD group was similar to the control group regarding sex, smoking status, alcohol, diabetes, hypertension, or hyperlipidemia in this population.

This study has several limitations, including a relatively small sample size. We did not perform a thorough analysis of AMD subtypes, such as geographic atrophy or early stage of AMD. This is because only wAMD participants who consented to treatment with anti-VEGF therapy were available for the AH collection. Additionally, this study comprised solely Asian participants, which may be related to epidemiologic factors of AMD. Further, investigating metabolic changes in AH does not necessarily reflect the real intraocular metabolism. Ideally, metabolic screening for retinal diseases should be obtained from vitreous specimens. Concerning clinical ethics and feasibility, harvesting AH before recruiting patients for intravitreal injection is a realistic approach. We plan to identify differences in metabolism between the AH, vitreous, and retina in an animal CNV study with metabolomics methods as the primary endpoint. Additionally, it would be interesting to analyze how other metabolic pathways are altered, including amino acid and fatty acid pathways related to glucose metabolism. Future AH metabolomic analyzes are underway for characterizing the AH profile. Finally, metabolites from AH are dynamic and affected by other factors, such as specimen preservation time and determination method. Longitudinal studies are required to confirm our results and to evaluate the evolution of metabolites with AMD progression.

## Conclusions

Our data provide a quantitative analysis related to glucose metabolism of AH in AMD, and highlight significant changes in the levels of glutamine, glutamate, and TCA-related organic acids, particularly *α*-ketoglutarate and citrate levels. The changes in phakic and pseudophakic groups were not significant, and only pyruvate was associated with the form of the crystalline lens. We also identified a higher K/C ratio in control patients with cataract and a lower K/C ratio in AMD participants. This ratio was correlated with glutamine in all groups, indicating perturbed reductive glutamine use in AMD pathogenesis. Short-term anti-VEGF treatment did not alter metabolite status in the current study. These findings offer potential metabolic targets for treatment of AMD, which may require modulating distinct metabolite concentrations rather than inhibiting a certain signaling pathway.

## Supplementary Material

Supplement 1

Supplement 2
