# Supplementary material for: Glucose Metabolic Characterization of Human Aqueous Humor in Relation to Wet Age-Related Macular Degeneration
Source: Invest Ophthalmol Vis Sci. 2020 Mar 30;61(3):49. doi: 10.1167/iovs.61.3.49 (PMC7401462; doi:10.1167/iovs.61.3.49)
Supplement: Supplement 1 [file iovs-61-3-49_s001.pdf]

Supplementary Figure 1. Quality control were performed to exclude metabolites with Quality Control Relative Standard Deviation (QC RSD) less than 30%.

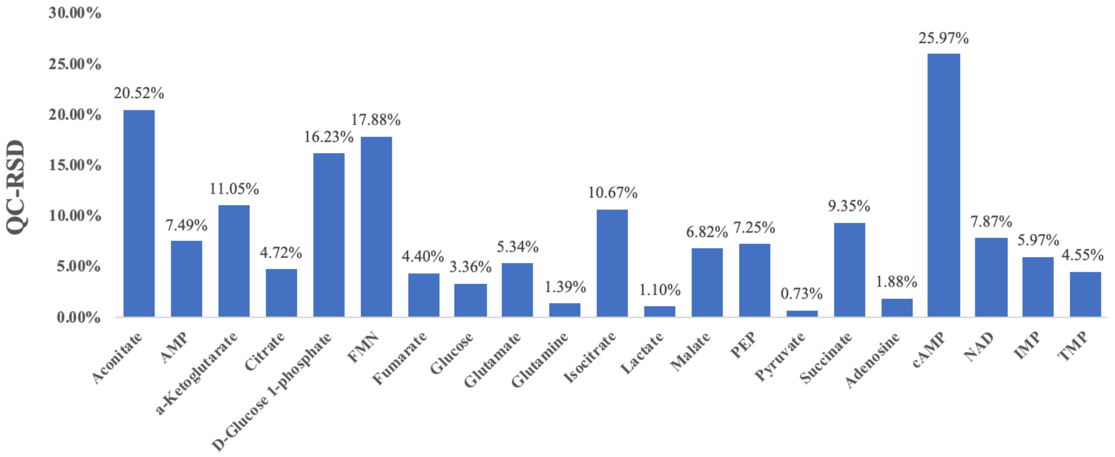

AMP: Adenosine monophosphate; FMN: Flavin mononucleotide; IMP:Inosine 5'-monophosphate; PEP: Phosphoenolpyruvate; AMP: Adenosine monophosphate; TMP: Thiamine monophosphate; NAD: Nicotinamide adenine dinucleotide.
