# Supplementary material for: Glucose Metabolic Characterization of Human Aqueous Humor in Relation to Wet Age-Related Macular Degeneration
Source: Invest Ophthalmol Vis Sci. 2020 Mar 30;61(3):49. doi: 10.1167/iovs.61.3.49 (PMC7401462; doi:10.1167/iovs.61.3.49)
Supplement: Supplement 2 [file iovs-61-3-49_s002.pdf]

Supplementary Table 1. A summary of parameters evaluated for performance assessment of UHPLC-MS/MS for targeted energy metabolites in positive and negative ionization MRM mode.

Component Name	MS Mode	RT (min)	Standard Calibration Curve	R^2
Aconitate	Neg	12.7	y=8392.7*x	0.9517
a-Ketoglutarate	Neg	10.5	y=3223.92*x	0.9993
AMP	Pos	12.1	y=2.00544e+006*x	0.9984
cAMP	Neg	5.19	y=2.52162e+008*x	0.9818
Citrate	Neg	12.8	y=1944.06*x	0.9233
D-Glucose 1-phosphate	Neg	12.5	y=19734.2*x	0.9991
FMN	Pos	11.7	y=417853*x	0.9984
Fumarate	Neg	11.1	y=23618.7*x	0.9988
Glutamate	Pos	11	y=221916*x	0.9998
Glutamine	Pos	10.5	y=493163*x	0.9912
IMP	Pos	12.3	y=159417*x	0.9937
TMP	Pos	16.57	y=211891*x	0.9993
Isocitrate	Neg	12.6	y=26922*x	0.9463
Lactate	Neg	5.3	y=31764.1*x	0.9996
Malate	Neg	11.1	y=262827*x	0.9974
Phosphoenolpyruvate	Neg	12.7	y=10953.3*x	0.9984
Pyruvate	Neg	1.8	y=1500.67*x	0.9621
Succinate	Neg	10.7	y=46529.6*x	0.9993
Glucose	Neg	8.4	y=141.45+0.438797*x	0.9994
NAD	Pos	13.92	y=546619*x	0.9775
Adenosine	Pos	4.8	y=3.41487e+006*x	0.9848

AMP: Adenosine monophosphate; FMN: Flavin mononucleotide; IMP:Inosine 5'-monophosphate; PEP: Phosphoenolpyruvate; AMP: Adenosine monophosphate; TMP: Thiamine monophosphate; NAD: Nicotinamide adenine dinucleotide.
